# Shedding Light on Filovirus Infection with High-Content Imaging

**DOI:** 10.3390/v4081354

**Published:** 2012-08-23

**Authors:** Gianluca Pegoraro, Sina Bavari, Rekha G. Panchal

**Affiliations:** 1 United States Army Medical Research Institute of Infectious Diseases (USAMRIID), 1425 Porter Street, Fort Detrick, MD 21702, USA; Email: gianluca.pegoraro.ctr@amedd.army.mil (G.P.); sina.bavari@amedd.army.mil (S.B.); 2 PerkinElmer, 940 Winter Street, Waltham, MA 02451, USA

**Keywords:** filoviruses, High-Content Imaging, therapeutics, host-pathogen interactions, phenotype

## Abstract

Microscopy has been instrumental in the discovery and characterization of microorganisms. Major advances in high-throughput fluorescence microscopy and automated, high-content image analysis tools are paving the way to the systematic and quantitative study of the molecular properties of cellular systems, both at the population and at the single-cell level. High-Content Imaging (HCI) has been used to characterize host-virus interactions in genome-wide reverse genetic screens and to identify novel cellular factors implicated in the binding, entry, replication and egress of several pathogenic viruses. Here we present an overview of the most significant applications of HCI in the context of the cell biology of filovirus infection. HCI assays have been recently implemented to quantitatively study filoviruses in cell culture, employing either infectious viruses in a BSL-4 environment or surrogate genetic systems in a BSL-2 environment. These assays are becoming instrumental for small molecule and siRNA screens aimed at the discovery of both cellular therapeutic targets and of compounds with anti-viral properties. We discuss the current practical constraints limiting the implementation of high-throughput biology in a BSL-4 environment, and propose possible solutions to safely perform high-content, high-throughput filovirus infection assays. Finally, we discuss possible novel applications of HCI in the context of filovirus research with particular emphasis on the identification of possible cellular biomarkers of virus infection.

## 1. Introduction

Fluorescence microscopy is used to study biological processes with high spatial and temporal resolution, and has become one of the techniques of choice to study viral infection in the context of the host cell. More recently, in the High-Content Imaging (HCI) field, technological advances related to assay miniaturization, high-throughput microscopy, and automated image analysis have made it possible to systematically acquire fluorescence microscopy images and to extract up to hundreds of functional and morphological features at the single-cell level [1,2]. Consequently, cell biology, which was traditionally semi-quantitative and based on a relatively small number of image based observations, can now effectively rely on the quantitation of molecular and phenotypic events at the system level. HCI is now used to address biological questions in a range of disciplines including oncology, *in vitro* toxicology, neuroscience, immune function and infectious diseases [3,4,5,6,7,8].

The emergence of new viruses, as well as the potential danger of misusing deadly hemorrhagic fever viruses or eradicated viruses as agents of bioterrorism, highlights the need to develop novel antiviral strategies that could aid in a rapid response scenario [9]. Filoviruses are the etiologic agents of severe hemorrhagic fevers with high-case fatality rates in infected humans and non-human primates [10]. These viruses are highly infectious and require Biosafety Level 4 (BSL-4) containment measures. Although clinically approved vaccines or therapeutics to treat filovirus disease are not available yet, significant progress has been made in the development of multiple candidate vaccines (reviewed in [11,12,13]) and therapeutics targeting filoviral genes [14,15]. 

In this review we discuss the impact, the challenges and the possible applications for HCI in filovirus research. First, we briefly describe the molecular biology of filoviruses and the existing recombinant filovirus genetic systems that can be used in imaging-based studies. We will then present an overview of HCI technology and how it can be applied to answer a wide variety of biological questions. Several examples of successful applications of HCI in studies of host-cell interactions will be presented. In particular, this review will focus on the use of cellular imaging for drug discovery screens and for the identification of novel host targets of viral infection. Finally, we will suggest possible novel applications of HCI for the study of filoviruses and their interaction with the host cell.

## 2. Filoviruses

Filoviruses are enveloped, single-stranded, negative-sense RNA viruses that cause severe hemorrhagic fever in humans and non-human primates, with case-fatality of up to 90% in humans [16]. After exposure, the onset of clinical signs and symptoms ranges from two days to as long as twenty‑one days; however, most infected patients succumb to disease in seven to ten days. The outbreaks are sporadic and have been restricted to rural and sparsely populated regions, or underdeveloped urban areas in Africa. The natural host reservoir for filovirus has not been definitely identified, although fruit bats have been implicated [17,18]. These viruses are considered to be biothreat agents, due to their high case fatality rate, person to person transmissibility, and potential aerosol delivery route [9]. Currently, there are no approved prophylaxis or post-exposure treatments for filovirus infections, and supportive care remains the only option for treating patients infected during filovirus outbreaks.

The family *Filoviridae* consists of three genera, *Ebolavirus* and *Marburgvirus*, and “Cuevavirus” (Tentative). There are two recognized marburgviruses, Marburg virus (MARV) and Ravn virus (RAVV). Five viruses are assigned to the genus *Ebolavirus*: Ebola virus (EBOV), Sudan virus (SUDV), Reston virus (RESTV), Taï Forest virus (TAFV), and Bundibugyo virus (BDBV). Only one “cuevavirus”, Lloviu virus (LLOV), has thus far been discovered [19,20,21]. The 19 kb RNA genome encodes for seven viral proteins: glycoprotein (GP), virion protein (VP) 24, VP30, VP35, VP40, nucleoprotein (NP) and RNA-dependent RNA polymerase (L protein) [22,23,24]. Although the virus genome encodes all the structural proteins required to make the virus particle, filoviruses like other enveloped viruses such as HIV-1, hijack components of the host cellular machinery for entry, trafficking, transcription, replication, assembly and budding of the virus from the cell membrane [25,26,27].

The molecular mechanisms of entry, replication, assembly and budding, as well as the modulation of cellular signaling pathways by viral proteins are areas of active research, for which details have been reviewed elsewhere [28]. Briefly, filovirus infection is initiated following attachment of the virions to multiple cell-surface molecules on the target cells [29,30,31]. Filoviruses exhibit diverse cell tropism, as they can infect a wide variety of cell types including innate immune cells such as dendritic cells, monocytes, macrophages and non-lymphocytic cell types during the late stages of *in vivo* infection. The viruses gain entry into the cell by endocytosis, which may occur via macropinocytosis and clathrin mediated endocytosis [32] or possibly via clathrin- and caveolin independent endocytosis [33]. The entry mechanism of filoviruses is yet to be fully characterized and it is conceivable that these viruses use various pathways depending on the cell type they infect. Following endocytosis, fusion of the viral envelope with the endosomal membrane results in uncoating and release of the nucleocapsids into the cytosol. The acidic pH in the endosomes, the proteolytic cleavage of the GP protein, and the cellular CTSB, CTSL and NPC1 proteins contribute to the fusion process [34,35,36,37,38]. The next step is the transcription of viral RNA and mRNA by the viral RNA polymerase and the translation of the viral proteins by the host machinery. After replication and encapsidation of the nascent viral genome, virus particles are assembled and bud from the cell membrane. A growing amount of evidence indicates that during the different phases of its replication life cycle the virus exploits host pathways such as protein transport and sorting (TSG101, NEDD4 and VPS4 are involved in this process [39,40,41,42,43]), the cytoskeleton machinery, and also protein post-translational modification cascades such as phosphorylation, glycosylation and ubiquitination [44]. In addition, during infection the virus inhibits the host interferon response, thus preventing the development of an antiviral state [45,46,47]. A better understanding of this complex network of host-virus interactions will facilitate the targeted design of antivirals against filoviruses.

## 3. High-Content Imaging (HCI)

HCI stems from the combination of high-throughput microscopy with automated, multiparametric image analysis. This novel technology has been employed during all phases of the drug discovery process and it is increasingly becoming available in academic settings for high-throughput cell biology studies [1,48]. HCI is a complex, multi-stage process and requires an integrated effort from various disciplines. As such, the HCI screening workflow comprises several critical steps that include assay development, automated image acquisition and analysis, secondary data analysis, visualization and management [49].

Depending on the application, establishment of a robust and validated HCI assay requires optimization of several variables: (i) Choice of cells and cell-related parameters: primary cells *versus* cell lines, adherent cells *versus* suspension cells, live (kinetic) or fixed (end-point) cell assays, plating density *etc.*; (ii) Choice of multi-well imaging plates: the choice of the multi-well imaging plate will often dictate the objective lens that can be used for imaging; (iii) Statistical validation requirements (sensitivity, dynamic range, signal intensity and stability) are very similar to those applied for any high-throughput screening campaigns; (iv) Detection reagents: primary antibodies, fluorescent secondary antibodies, and fluorescent cellular dyes.

In an HCI assay biological processes can be visualized by expressing a genetically encoded fusion of a fluorescent protein (such as GFP or any of its spectral variants) to a relevant cellular target of choice [50]. Alternatively, standard immunofluorescence (IF) protocols can be used to label the endogenous protein of interest [51], or to detect protein post-translational modifications such as phosphorylation by using phospho-specific antibodies. Specific fluorescent dyes (such as Hoechst33342, CellMask, Mitotracker, *etc.*) can also be used as markers to detect specific subcellular compartments or responses to chemical changes in the cellular environment (such as pH or an increase in oxidative conditions, *etc.*). Changes in the sub-cellular localization relative to a landmark of interest, as well as spatial arrangement and/or protein expression levels are all properties of the fluorescently labeled marker that can be used as a quantitative readout of biological activity [2,52]. It is also possible to conduct fluorescent label-free experiments by phase contrast light microscopy in which the marker of interest is cellular morphology itself, as defined by a combination of size-, shape- and texture‑related cellular features [3].

In a typical HCI assay workflow, cells are first seeded in multi-well clear bottom imaging plates (96-, 384- or 1536-well plates are the currently available standards). Robotic liquid handling equipment is then used to add libraries of thousands of small molecules or siRNAs to the cells. In the case of wide-field microscopes, the excitation light is provided by either a xenon or mercury lamp and the appropriate wavelengths are selected by a set of excitation filters. In confocal microscopy instead, the excitation source is provided by a laser that can be focused on a specific imaging plane in the vertical dimension, thus eliminating the interference of out-of focus light during detection. In either case, high-throughput microscopes generally employ high-speed CCD cameras to collect and quantify fluorescence from the illuminated samples. Since all the steps necessary for image acquisition (stage movement, focusing, fluorophore excitation and fluorescence detection) are automated, high‑throughput microscopes can produce two dimensional fluorescence images of cells from multiple channels, multiple fields within a single well and multiple wells in the same plate. HCI microscopes equipped with environmental chambers can also be used for live cell imaging to study dynamic processes in real time. As an example of its possible applications to the study of viruses, real-time imaging in living cells of recombinant viral RNA using a fluorescent protein-tagged version of the MS2 RNA binding protein has been extremely useful to quantitatively study and model transcription and replication of human immunodeficiency virus 1 (HIV-1) and hepatitis C virus (HCV) [53,54,55]. Depending on the cellular assay of interest and on the acquisition parameters, HCI studies can produce hundreds of thousands of fluorescence images per day.

**Figure 1:**
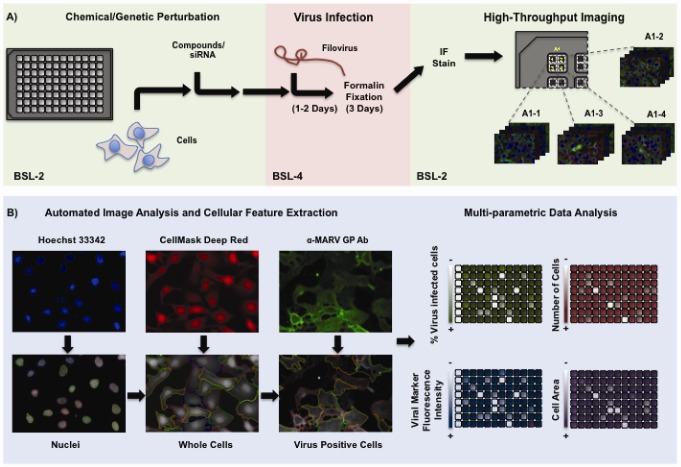
(**A**) Schematic representation of the experimental phases of an HCI infection assay using a wild-type filovirus. The Biosafety Level (BSL) at which the different steps of the assay are performed are indicated in the figure. (**B**) The different steps of the analysis (Image segmentation, cellular feature extraction, and detection of filovirus infected cells) of 2D fluorescence microscopy images are indicated. The analysis provides multiple experimentally relevant outputs in a medium- to high-throughput.

Dedicated image analysis software is then used to rapidly extract and quantify multiple cellular features of interest from thousands of acquired images in an automated and unbiased fashion [2,52]. Depending on the type of imaging instrument, image analysis can also be performed “on-the-fly”, as the images are being acquired. Images are first illumination-corrected to take into account possible artifacts due to an uneven distribution of the excitation light across the imaging field. Based on the pixel intensity distributions in the image, the analysis software calculates a threshold, which is then used to separate the background from the foreground. The foreground is further segmented in order to identify the cellular objects such as nuclei, cell membranes or spots. In order to discriminate between different cells, nuclei are generally segmented first because in most cases they are separated from each other and a one-to-one relationship exist between the number of cells and the number of nuclei. In addition, nuclear shape tends to be round and homogeneous, and hence computationally easier to recognize when compared with other cellular structures. Identified nuclei are used by the software as the starting point to further identify secondary interesting cellular regions in the cells, such as the plasma membrane edges and the cytoplasm (Figure 1). Once the cellular regions of interest are defined, the software can calculate values associated with multiple features of the segmented objects such as number, position, morphology, fluorescence intensity, and texture. An image analysis routine can output up to several hundreds different features at the single-cell level.

Various statistical methods and tools are applied to normalize and reduce the complexity of the multi-parametric cellular feature dataset [56,57]. In addition, machine learning algorithms have been developed for the automatic classification of cell subpopulations based on the phenotypic profile generated from the feature dataset [58,59,60]. This is particularly useful when dealing with heterogeneous populations of cells and for the clustering of treatments based on their effect on cellular morphology [61,62]. Thus, if treatment of cells by siRNA or small molecules results in unexpected and/or complex phenotypes, the phenotypic cellular fingerprint generated by HCI analysis can be used to detect a biological response. HCI has been used to identify the mechanism of action, the cellular process targeted, and possible cytotoxic or off-target effects of the chemical or genetic perturbation under exam [61,63].

## 4. HCI in the Context of High-Throughput Virus Research

Improvements in RNAi technology have made it possible to specifically silence gene activity on a genome-wide basis in mammalian cells. Functional, reverse genetic screens harnessing HCI have been useful to investigate gene function in a wide range of biological processes such as the DNA damage response [64,65,66], mitosis [67], alternative RNA splicing [68], autophagy [69] and endocytosis [70]. HCI has also been widely applied to systematically study host-virus interactions in chemical and genetic screens [7]. In fact, targeting host genes for anti-viral therapy holds the promise to expand the range of possible therapeutic targets beyond the limited number of viral gene products, and may also help overcome the issue of drug-resistance due to the fast mutation rate of viral genomes [71]. HCI is particularly suited to quantify viral infection in cell culture because it can be used to identify and analyze heterogeneous subpopulations of cells (virus infected *versus* uninfected cells, as an example) within the same well. In addition, cytotoxic compounds or siRNAs can be rapidly filtered out in an HCI assay by measuring the number of cells present at the end of the treatment in each well.

HCI-based assays have been used to conduct genome-wide siRNA screens for the systematic identification of host genes that modulate the replication of HIV-1 [72,73], West Nile virus [74], HCV [75] and influenza A virus [76,77] in mammalian cells. In all these genome-wide siRNA screens cells were first pretreated with siRNA, infected with the virus and then stained with an antibody recognizing a specific viral antigen. The percentage of infection in each experimental condition was then calculated based on the number of cells showing a positive fluorescence signal associated with the viral marker. By analyzing the percentage of infected cells following a single infection cycle, it was possible to identify genes whose knock-down inhibited virus binding, entry, transcription/replication, translation, or trafficking. In two of these genome-wide screens [73,76], defects in virus egress were also evaluated by a second round of infection in which supernatants of siRNA-treated and virus infected cells were transferred onto cells expressing a non-HCI reporter of virus infection. As a result, the authors were able to identify hundreds of cellular genes necessary for viral infection. As an alternative approach to the use of infectious viruses, it is also possible to use subgenomic replicon‑based systems to identify genes involved in viral RNA replication, as in the case of HCV [78].

One of the major caveats of these studies is the poor, albeit significant, overlap of the hit gene lists obtained from independent genome-wide siRNA screens for the same virus [79]. As for any genome‑wide siRNA screen, both high false-positive and high false-negative rates need to be taken into account when interpreting results due to possible siRNA off-target effects [80]. Additionally, it is generally assumed that the cell lines routinely used for siRNA screens are homogenous, and that all the cells subject to the same treatment should show similar biological responses. For this reason, and as it is routine for most other siRNA screens, the effect of gene knock-down on virus infection in the above mentioned studies was measured by averaging the percentage of infected cells on a per-well basis. However, it is becoming increasingly clear that even monoclonal mammalian cell lines show a substantial degree of heterogeneity [81,82,83]. Single-cell HCI analysis and data modeling have revealed that population context exerts an important influence on the metabolic state, the endocytic activity, and, more importantly, on the permissiveness of cells to virus infection [81]. Further studies also indicate that the presence of subpopulations of cells which differ in their biological status is one of the major sources of variability when analyzing the results of siRNA screens against virus infection [84,85]. Importantly, by using single-cell based statistical models it is possible to normalize the variability associated with population context. This normalization step increases the specificity and the robustness of the results obtained in several siRNA screens aimed at the identification of host factors required for virus infection [84,85].

HCI is also being currently applied for the discovery and characterization of therapeutic small molecules for the treatment of infectious diseases [7]. One significant obstacle to such cell-based chemical screens is the identification of the therapeutic target of candidate compounds. To partly address this issue, large number of morphological features extracted from HCI small molecule screens, can be used to generate phenotypic profiles and infer the biological target of the tested small molecule [63]. Hence, HCI-based assays have the potential to provide useful information to profile anti-viral compounds with unknown mechanism of action according to their phenotypic effect on cells. Robust HCI assays for the discovery of compounds with antiviral activity have been already optimized for Dengue-2 virus (DENV-2) and HCV [86,87].

## 5. Challenges in the Implementation of HCI for BSL-4 Pathogens

One of the challenges encountered in developing effective medical countermeasures for filoviruses is the lack of suitable high throughput assays for screening chemical libraries [88]. Traditional viral plaque assays are often used to quantitate infectious filovirus particles in biological samples [89]. However, this method is time consuming and not amenable for high-throughput screening. Real time PCR is an excellent alternative for the quantitation of filoviral RNA transcripts to test the effects of antiviral compounds [90,91]. However, this method cannot discern infectious from defective virus particles and/or if antiviral activity of the compound was due to cytotoxicity. Quantitation of virus‑induced cytopathic effects is another assay that could be useful for screening chemical libraries. However, it would be difficult to find very effective drugs that can protect cells during the late stage of infection and hence this method is not suited as a primary screening assay.

Due to the biosafety concerns related to the handling of filoviruses, several surrogate models have been developed to study filovirus lifecycle events in a BSL-2 laboratory. The non-infectious virus like particle (VLP) release assay was developed to study assembly and release of filoviral particles [92,93]. VLP systems are based on the filovirus matrix protein (VP40), which is the driving force of filovirus budding. Accessory viral proteins such as GP and NP are also often included. A surrogate system to study filovirus cell entry consists of retroviral vectors (most commonly Vesicular stomatitis virus or Moloney murine leukemia virion-like particles) that carry the filoviral GP spike complex in their envelope and that encode a reporter gene, such as eGFP or luciferase, to facilitate quantification of transduction efficiencies. This pseudotype system is feasible because enveloped viruses can incorporate heterologous viral GPs into their lipid membranes during budding and because filovirus cell attachment and entry are mediated exclusively by the envelope GP spike complex. VSV or HIV viruses pseudotyped with EBOV-GP and expressing a luciferase reporter have been successfully used in high-throughput screening campaigns that lead to the identification of filovirus entry inhibitors [36,94,95]. Minigenome or minireplicon systems have also been established for filoviruses by utilizing reverse genetic techniques. These systems enable studies of filovirus transcription and replication [96].

Given the extremely hazardous nature of these pathogens several logistical challenges have been encountered while setting up high-throughput assays for infectious filoviruses. A primary concern is the generation of aerosolized virus during dispensing. Furthermore, the limited availability of physical space in BSL-4 containment suites makes it difficult to implement large robotic liquid handling workstations that are typically required for a high-throughput workflow. The lack of viable automated liquid handling solutions constitutes the main bottleneck in the HCI filovirus infection assay workflow, thus currently limiting its implementation to a 96-well format, rather than the more resource- and time‑efficient 384-well format. The feasibility of performing viral infections in 384-well imaging plates has already been shown for BSL-2 pathogens [73,74,75,77,85,87]. In order to overcome the undesired and potentially dangerous production of aerosolized virus, it will be worth considering the possibility of first safety-testing and then implementing small footprint safety cabinets containing filter-tip based, automated liquid handling instrumentation to achieve high-throughput dispensing of BSL-4 pathogens. Finally, another possible hurdle for HCI assays in a BSL-4 setting is that, to inactivate the virus, infected cells must be treated with 10% formalin for three days before bringing the samples out of the containment suites [97]. These harsh fixation conditions can hamper the efficiency of IF staining procedures and thus require careful validation of all antibodies during the assay development phase.

Despite these issues, carefully designed HCI-based assays can be effectively optimized and implemented to comply with necessary safety concerns associated with handling of BSL-4 pathogens [97]. The general workflow of a typical HCI assay to measure filovirus infection is depicted in Figure 1. High-throughput cell seeding and dispensing of the perturbing reagents (small molecule or siRNA) is performed in a BSL-2 environment in a 96-well format (Figure 1A). Treated cells are then transferred to BSL-4 suites, where they are infected for the appropriate amount of time with filoviruses. At the end of infection, cells are fixed for at least 72 hours in formalin. Fixed cells are then subjected to the IF staining protocol in a BSL-2 laboratory using standard liquid handling automation. After this step, the appropriate fluorescent dyes labeling the nucleus and the plasma membrane are added. Automated image acquisition and analysis is performed as previously described (Figure 1B). Nuclei are segmented based on the Hoechst 33342 DNA stain and used as the nucleating object to then expand a cytoplasmic mask. Cytoplasm boundaries are determined with the CellMask Deep Red cell membrane stain and the fluorescence signal for the viral marker is quantified. The efficiency of filovirus infection following exposure of cells to different perturbing agents, is then calculated based on the percentage of cells positive for the viral IF stain, or for the green fluorescent protein (GFP) if using a recombinant EBOV engineered to express enhanced GFP (EGFP) [97]. The analysis also outputs a range of other cellular parameters, such as the number of nuclei per well, the mean cellular area and the mean intensity of the fluorescent marker.

To successfully implement HCI assays for filoviruses, both biological assays parameters and image acquisition and analysis parameters needed to be optimized [97]. Cell seeding conditions, filovirus MOI, and the duration of viral infections were optimized to reproducibly reach high rates of infection (60% for MARV and 75% for EBOV-EGFP). These optimization experiments revealed that to achieve a robust filoviruses infection rate and high Z' scores, cells were required to be seeded at high-density (30,000 or 40,000 Vero cells/well in a 96-well format) and infected for 48 or 72 hours with 5 or 20 MOI of EBOV-GFP or MARV, respectively. Under these experimental conditions, the cells formed a continuous monolayer, which made it difficult to accurately segment them during the image analysis phase. A precise image analysis protocol was developed for the accurate nuclear and cell segmentation of tightly packed cells [97]. As a result of these careful optimization steps, the Z' scores for the EBOV‑GFP and the MARV based HCI assays were 0.8 and 0.75, respectively, and the Signal‑to-noise ratio approached the value of 20 in both cases [97]. These results demonstrated that it is possible to perform HCI assays for BSL-4 pathogens with quality metrics comparable to other HCI assays optimized to measure infection of other BSL-2 viruses, such as DENV-2 (Z' = 0.52, Signal-to-noise ratio = 14 [87]). Assays optimized for both EBOV and MARV infections were then applied to identify small-molecule inhibitors of filovirus infection [97,98,99]. As a proof of principle, the HCI-based assay was used to screen 1900 compounds belonging to the NCI diversity library. This screen led to the identification of NSC 62914, a small molecule with antioxidant properties that has potent, wide‑spectrum antiviral activity and that can protect mice from EBOV in a post-challenge model of infection [98]. Further evidence of the usefulness of HCI in identifying anti-filoviral therapeutic targets was obtained in a proteomics study of host proteins associated with EBOV and MARV particles. The functional role of the identified proteins in EBOV and MARV infection was validated by using a HCI‑based siRNA screen [100]. An HCI-based assay was used to measure the effect of the BST‑2/Tetherin protein on filovirus infection in cell culture by either overexpressing or knocking‑down the expression of this cellular gene [101]. In addition, HCI assays using MLV-EGFP particles pseudotyped with MARV GP, MARV or EBOV-EGFP have been employed to test the inhibitory potential on filoviral infection of Δ-peptides produced by lethal filoviruses [102]. Altogether, the development and implementation of these HCI assays constitute the first successful attempt at quantitatively measuring replicating filovirus infection in a medium to high throughput fashion for the identification of anti-viral compounds and/or gene products that modulate filoviral infection.

## 6. Future Applications of HCI for Filovirus Research

Major advances in image-based screening technologies have already greatly benefited the study of infectious diseases and in particular of filoviruses. However, in order to achieve a system view of the cellular responses to infection in presence or absence of the perturbing agents, the multi-parametric dataset generated from various HCI-based filovirus infection assays should be fully harnessed. In particular, statistical analysis of single-cell image analysis data should be used to normalize the effects of cellular heterogeneity in the population of filovirus infected cells [84,85]. Furthermore, data at the single-cell level could also be analyzed to measure spatial clustering of viral infected cells [103]. This property can be used to infer the biological effect of a specific perturbation (whether siRNA or small molecule) on a particular phase of the virus life cycle, such as virus replication (size or area of foci of infected cells) or virus spread (Number and distance of the foci of infected cells).

HCI-based filovirus screens are generally carried out in cell lines such as African green monkey VeroE6 cells, which are typically used for large-scale production of these viruses, or in human Hela or HEK293 transformed cells, which can be easily transfected by oligo siRNA. However, HCI assays that employ more physiological cellular targets of filovirus infection, such as primary monocytes and macrophages [104], should be considered and optimized, at least for more focused and biologically-relevant secondary screens [105].

Finally, a largely unexplored and potentially interesting area of investigation in filovirus research is the use of HCI for the systematic study of host-virus interactions, for the discovery of cellular biomarkers and for the development of molecular signatures of infection. Several HCI assays could be used in parallel to measure changes in the activity of relevant cellular signaling pathways, such as inflammation, cellular stress and DNA damage [106]. While this approach is more laborious when compared with the extraction of purely phenotypic cellular measurements, the use of specific signaling markers simplifies the identification of the molecular mechanisms linking a perturbation (such as infection with a specific pathogen, for example) with its observed cellular effects. The application of an array of 50 HCI assays to measure the activity of a broad panel of cellular signaling pathways was successful in characterizing hidden phenotypes and the mechanisms of action of a library of 107 active compounds [107]. A similar approach could be applied in parallel infection assays with several filoviruses to detect the different cellular pathways modulated by filovirus infection. The activation or the inhibition of a certain cellular pathway upon filovirus infection could then be used as a biomarker. More importantly, the integrated analysis of the responses of several cellular signaling pathways to infection with a specific pathogen could then be applied to build a signaling-based fingerprint for the functional clustering of viruses based on host cell responses.

In summary, HCI-based assays have been proven to be: (1) adaptable to strict BSL-4 containment conditions; (2) capable of quantitatively and reproducibly measuring filovirus infection in a medium- to high-throughput format; and (3) the method of choice for the screening of large libraries of small molecules for the discovery and characterization of antiviral therapeutic compounds. The application of novel technological advances in HCI will help gain a better understanding of basic filovirus biology at the system level, and in the identification of effective medical countermeasures against filovirus infection.
